# Multiparametric quantitative and texture ^18^F-FDG PET/CT analysis for primary malignant tumour grade differentiation

**DOI:** 10.1186/s41747-019-0124-3

**Published:** 2019-12-18

**Authors:** Mykola Novikov

**Affiliations:** Israeli Oncologic Hospital LISOD, 27 Malyshko str., Pliuty, Obuhovskiy district, Kyiv region, 08720 Ukraine

**Keywords:** Biomarkers, Fluorodeoxyglucose F18, Radiomics, Positron emission tomography computed tomography, Texture analysis

## Abstract

**Background:**

^18^F-FDG positron emission tomography/computed tomography (PET/CT) is a successfully used imaging modality in oncology. The aim of the study was to investigate a connection of epithelial tumour differentiation grade with both semiquantitative and quantitative metabolic PET data focusing on creation of multiparametric model of tumour grade prediction utilising both standardised uptake value-based and texture-based ^18^F-FDG PET parameters and to investigate an influence of different image segmentation techniques on these parameters and modelling.

**Methods:**

^18^F-FDG PET/CT data from 84 patients with epithelial malignant tumours was retrospectively analysed to create sets of both conventional semiquantitative (based on standardised uptake values), volumetric, and quantitative texture metabolic parameters of primary tumours with four different segmentation techniques.

**Results:**

Most of the calculated volumetric and texture parameters showed to be influenced by segmentation technique. There was no significant difference in values of only three parameters, in all four segmentation methods: homogeneity, energy, and sphericity. Almost every extracted parameter in all segmentation technique subsets showed significant ability to discriminate individual tumour grade *versus* the subset of remaining two tumour grades. No parameters were able to discriminate all three tumour grades separately simultaneously or without the overlapping of threshold values. Group method of data handling (GMDH) modelling included all the above-mentioned extracted parameters. The highest value to discriminate tumour grade was achieved using ITK-SNAP segmentation, with an accuracy ranging from 91 to 100%.

**Conclusions:**

Multiparametric modelling with GMDH utilising both semiquantitative and quantitative texture metabolic PET parameters seems to be an interesting tool for non-invasive malignant epithelial tumours grade differentiation.

## Key points

- Malignant tumour grade is an important prognostic factor.
- Metabolic positron emission tomography/computed tomography quantitative analysis showed ability to discriminate different tumour grades in primary epithelial malignant tumours.
- The highest accuracy in tumour grade prediction was achieved when multiparametric modelling is applied.

## Background

Metabolic positron emission tomography, combined with computed tomography (PET/CT) utilising ^18^F-labelled fluorodeoxyglucose (^18^F-FDG), is a successfully used imaging modality for oncologic patients in different clinical scenarios, ranging from staging to response assessment and prognostication [1–3]. Images produced by ^18^F-FDG PET/CT may be analysed visually or qualitatively by a physician and semiquantitatively or quantitatively by a vast range of parameters and metrics, derived from those images. Qualitative analysis by visual assessment may be sufficient for many routine oncological scenarios, but it is inevitably subjected to intra- and inter-observer variations [4].

True quantitative parameters in PET/CT are obtained by applying proper kinetic modelling to dynamic imaging acquisition, which is rarely performed in clinical practice. In daily work, to achieve objective interpretation, multiple semiquantitative parameters are generated, most of them being standardised uptake value (SUV) based and not requiring dynamic acquisition. Their clinical value was vastly studied and validated across multiple studies [5–10]. Along with objectification of PET data interpretation to support clinical decision-making, quantitative analysis provides new multiple layers of information that helps non-invasive tumour characterisation. Different methods of mathematical image manipulations, including texture analysis, were developed to extract multiple quantitative features from metabolic PET images.

The process aimed at mining of maximum amount of data from digital medical imaging eventually has been termed “radiomics”. Further combination of data acquired through radiomic process with different sorts of patient data (for example, clinical or laboratory tests, histopathological features, or genetic information) with bioinformatics tools allows to develop models that may potentially improve diagnostic, prognostic, and predictive accuracy [11].

Recently, multiple studies were conducted to search for imaging parameters as imaging biomarkers, resulting in creation of myriad of indices. Unfortunately, those indices had frequently discordant clinical value or relatively low repeatability as reported by different authors and groups. These limitations most likely arose due to multiple steps required to extract and calculate parameters, high dependence on image acquisition techniques, and variability of mathematical tools used to connect imaging metrics and clinical data [12].

In this study, we aimed to investigate the relation between metabolic ^18^F-FDG PET data (semiquantitative SUV-based and quantitative texture parameters) with tumour differentiation grade (as a basic parameter reflecting biologic aggressiveness) to generate a multiparametric model of tumour grade prediction and investigate about the influence of different image segmentation techniques on parameters and final modelling.

## Methods

### Patients

We analysed data from pre-treatment ^18^F-FDG PET/CT scans of 84 patients with primary epithelial malignant tumours, 44 males (53%) and 40 females (47%), with median (interquartile range) age of 56.5 (30–66) years: 21 (25.0%) with squamous cervical carcinomas, 30 (35.7%) with squamous head and neck carcinomas, and 33 (39.3%) with non-small cell lung carcinomas. All primary tumours were proven by histological examination of biopsy samples or surgical materials. All patients included did not have significant liver disease or liver failure at the moment of pre-treatment scan, according to laboratory exams. Differentiation grade of primary tumour lesions was identified during these examinations by pathologists with at least 7 years of experience according to the American Joint Committee on Cancer staging manual [13]. Grade 1 was identified in 8 tumours (9.5%), grade 2 in 50 (59.5%), and grade 3 in 26 (31%).

### PET/CT procedure

All ^18^F-FDG PET/CT scans were performed with a Gemini 16 PET/CT scanner (Philips Medical Systems, Cleveland, OH, USA). The examination technique was performed following previously published guidelines for ^18^F-FDG imaging in solid malignant tumours issued by the European Association of Nuclear Medicine (EANM) [14]. All patients fasted for 6 h prior to examination, blood glucose level before the ^18^F-FDG injection did not exceed 150 mg/dL, and time of ^18^F-FDG distribution varied from 60 to 75 min. The injected activity was calculated according to previously published guidelines [14]. The protocol included CT scanning after intravenous injection of iodinated non-ionic contrast agent Ultravist 370 (Bayer AG, Germany), with doses of 1 ml per 1 kg of patient’s weight in portal-venous acquisition phase and oral administration of water for better differentiation of bowel loops.

### Segmentation techniques

Four different techniques were applied to segment primary tumour volume on PET/CT images. A large spherical volume of interest (VOI) was initially manually placed to incorporate the whole visible tumour with additional caution paid not to include areas of high physiologic activity (such as urine in bladder for cervical tumours or myocardial activity for lung tumours) using multiplanar reconstructions. Segmentation inside this initial VOI was performed with four different methods or rules: fixed thresholding with SUVmax 2.5 cutoff; fixed thresholding with liver pool cutoff; fixed thresholding with 41% of SUVmax inside volume cutoff; and segmentation with the free, open-source ITK-SNAP software, version 3.8.0 (http://www.itksnap.org/pmwiki/pmwiki.php). The first three segmentation techniques were performed by OsiriX MD software, version 8.0.2 (https://www.osirix-viewer.com/osirix/osirix-md/).

When performing liver pool thresholding technique, the value of liver pool uptake was identified as mean SUV inside a spherical volume of interest (diameter 3 cm) placed in the right liver lobe, avoiding malignancies and organ boundaries, as previously suggested by published guidelines [14]. As already said, all patients did not have significant liver disease or liver failure at the moment of pre-treatment scan, according to laboratory exams.

The ITK-SNAP segmentation implements two 3D active contour segmentation methods: Geodesic Active Contours and Region Competition. Detailed mathematical insight into algorithms implemented in this software lies beyond the scope of current study and may be found in the paper by Yushkevich et al. [15]. Although initially designed and tested for anatomical segmentation of brain structures, this segmentation technique has been validated both for other anatomical segmentation applications, *e.g.,* for airway volume measurement on cone beam CT images by Almuzian et al. [16] and for lung cancer metabolic volume segmentation on ^18^F-FDG-PET imaging by Besson et al. [17].

### Semiquantitative and quantitative features extraction

All segmented tumour volumes are exported to LIFEx software, version 4.70 (https://www.lifexsoft.org/), for further semiquantitative and texture analysis. Several groups of semiquantitative parameters and texture features were extracted from segmented volumes. The conventional volumetric parameters were SUVmean, metabolic tumour volume (MTV), and tumour lesion glycolysis (TLG). Histogram parameters were skewness, kurtosis, entropy, and energy. Shape parameters were sphericity and compacity (the latter being the volume fraction that is filled in a granular medium, *e.g*., sand). Texture parameters were extracted from three different matrices: homogeneity and entropy from grey level co-occurrence matrix (GLCM); short-run emphasis (SRE) and long-run emphasis (LRE) from grey level run length matrix; and low grey level zone emphasis (LGZE) and high grey level zone emphasis (HGZE) from grey level zone length matrix.

Before texture feature extraction, spatial resampling, intensity rescaling, and intensity discretisation of segmented voxels were performed. All volumes were resampled to produce isometric voxels of 4 × 4 × 4 mm in size; absolute resampling was used for intensity rescaling with bounds from 0 to 30 SUV; and 64 grey levels applied for intensity discretisation. It should be noted that only tumour volumes bigger than 64 voxels were included into analysis to allow adequate texture features extraction, thus scans of the patients with relatively small tumours were initially excluded before the study group formation.

### Data analysis and multiparametric modelling

Statistical analysis of extracted data was performed with SPSS Statistics 21.0 software (IBM, Armonk, NY, USA). Initially, search for difference in values of collected quantitative features depending on segmentation technique was applied. As a second step, receiver operating characteristic (ROC) analysis of individual quantitative and texture parameters in order to discriminate tumour grade was performed. Following analysis of individual parameters, a multiparametric modelling utilising group method of data handling was used.

Values of individual parameters from different segmentation techniques were compared using multiple comparison with Bonferroni correction. Student’s *t* test and Mann-Whitney *U* test were utilised. Statistical test was chosen according to the type of data distribution, which was defined using Shapiro-Wilk *W* test. Data are presented as mean ± standard deviation (SD) or median and interquartile range (IQR), accordingly.

Group method of data handling (GMDH) algorithm, or also known as polynomial neural networks or abductive and statistical learning networks, was used to create predictive models.

GMDH is a set of several algorithms for solution of different modelling problems. It consists of parametric, clusterisation, analogue complexing, rebinarisation, and probability algorithms. This inductive approach is based on sorting out of gradually complicating models and selection of the optimal solution by minimum of external criterion characteristic. Initially suggested in 1971 by Ivakhnenko [18], this algorithm was developed and implemented in multiple practical scenarios, including handling of biomedical data [19, 20]. GMDH analysis was performed with dedicated GMDH DS software (GMDH LLC, USA, New York) version 6.4.

In order to compare and select the most powerful model, external criteria are generated with random separation of whole dataset into subsets. Parameter evaluation and assessment of model quality is based on different subsets. For this study, the primary dataset was divided into three parts. Models of different complexity were generated on teaching subset (70% of cases), and external criteria for choosing the optimal model were generated from exam subset (20% of cases). Additional 10% validation subset was generated to test the quality of generated model. This subset was not included in the process of model generation and selection.

Several measures were used to assess model performance during modelling process. For each model, C-statistic (similar to the area under the receiver operating characteristic curve), overall performance, root mean square error (RMSE), and F-measure were performed, along with sensitivity and specificity measures as most common measure to assess model performance.

## Results

### Influence of segmentation techniques on quantitative and texture indices

**Fig. 1:**
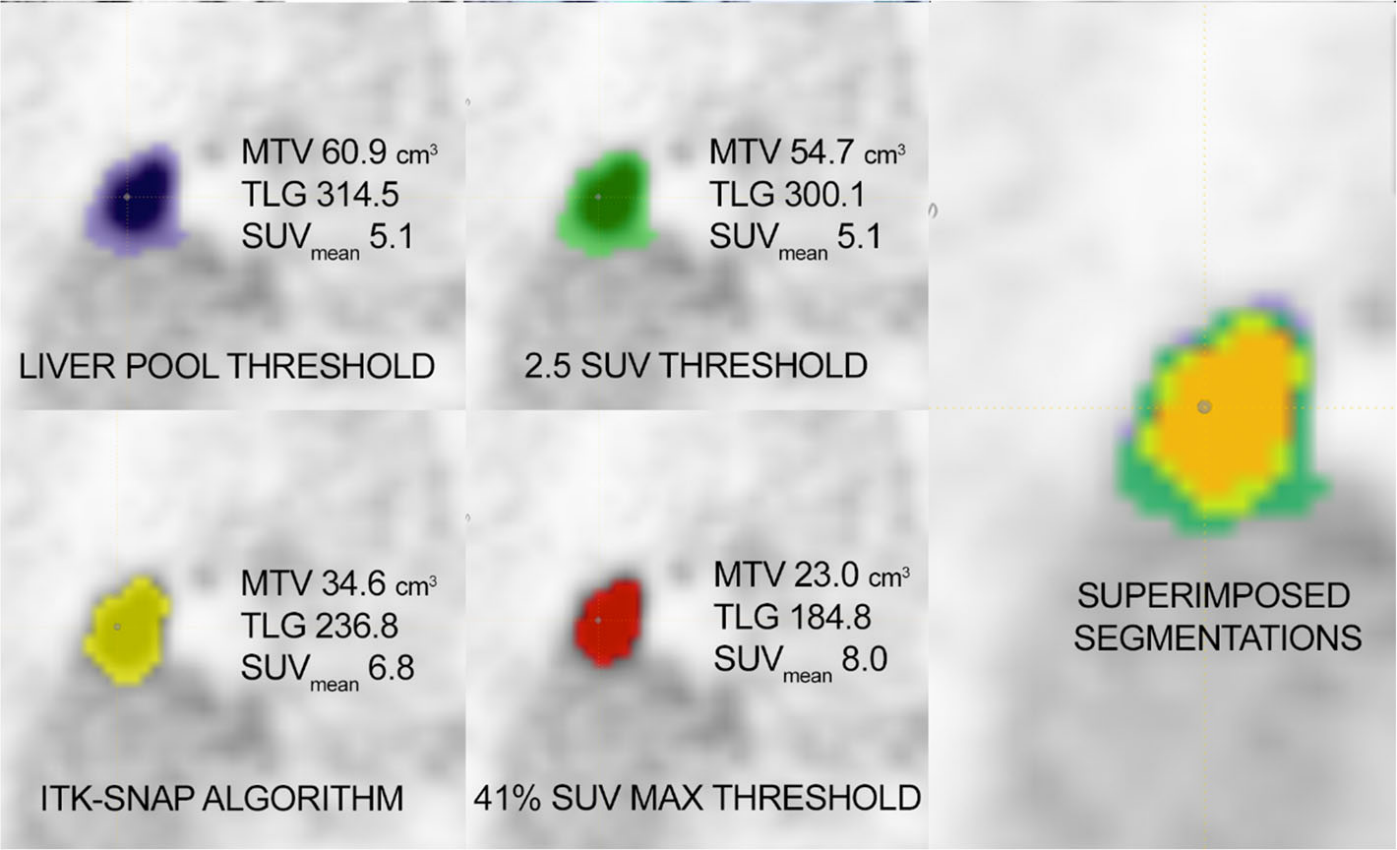
Different volumes of interest on PET images of a primary lung adenocarcinoma, produced by different segmentation techniques (presented with different colours), resulting in different values of semiquantitative metabolic parameters. *MTV* Metabolic tumour volume, *PET* Positron emission tomography, *TLG* Total lesion glycolysis, *SUV*mean Mean standardised uptake value

Most of the calculated indices showed significant difference in values, depending from segmentation technique (Fig. 1). There was no significant difference in values for only three parameters, in all four segmentation methods: homogeneity from GLCM, energy from histogram indices, and sphericity from shape analysis (*p* > 0.05 in all combination of pairs of segmentation techniques, Table 1). Conventional volumetric parameters (SUVmean, MTV, and TLG) showed significant variability, depending on segmentation technique, as being directly influenced by the number and value if segmented voxels. For instance, MTV values for different techniques varied as follows: median 38.7 (IQR 20.3–70.2) for SUVmax with 2.5 threshold; 19.6 (9.0–31.0) for 41% SUVmax; 53.0 (26.1–92.1) for liver pool fixed threshold, and 30.0 (16.6–54.5) for ITK-SNAP. There was no significant difference for all three conventional volumetric parameters between SUVmax with 2.5 fixed threshold technique and liver pool fixed threshold technique (SUVmean, *p* = 0.201; MTV *p* = 0.163; TLG, *p* = 0.393).

**Table 1:** Stable radiomic parameters, independent from segmentation technique

	GLCM homogeneity	Energy	Sphericity
Absolute values in different segmentation techniques			
SUVmax 2.5 threshold	0.37 ± 0.09	0.08 (0.06–0.12)	1.03 ± 0.05
Liver pool fixed threshold	0.38 ± 0.09	0.08 (0.06–0.12)	1.03 ± 0.04
41% SUVmax threshold	0.37 ± 0.08	0.09 (0.07–0.13)	1.04 ± 0.06
ITK-SNAP segmentation	0.37 ± 0.09	0.08 (0.06–0.13)	1.04 ± 0.05
*p* values for hypothesis of significant difference			
2.5 *versus* liver	0.582	0.632	0.844
2.5 *versus* ITK-SNAP	0.788	0.954	0.541
2.5 *versus* 41%	0.968	0.344	0.534
Liver *versus* 41%	0.554	0.122	0.633
Liver *versus* ITK-SNAP	0.423	0.596	0.653
41% *versus* ITK-SNAP	0.819	0.303	0.932

Data are presented as mean ± standard deviation or median with interquartile range in parentheses

*GLCM* Grey level co-occurrence matrix, *SUV* Standardised uptake value

### Individual quantitative and texture parameters and grade differentiation

Almost every extracted parameter in all segmentation technique subsets showed statistically significant ability to discriminate certain individual tumour grade (*versus* the subset of remaining two tumour grades, taking into account all possible pairs/combinations) at ROC analysis. But none of the parameters were able to discriminate all three tumour grades separately simultaneously or without the overlapping of threshold values. In separation of grade 1 tumours, the highest value was shown for MTV in SUVmax with 2.5 threshold and ITK-SNAP segmentation technique subsets with an area under the curve (AUC) at ROC analysis of 0.773 and 0.766 respectively. Applying a threshold of < 26.9, sensitivity was 81.0% and specificity 70.6%; applying a threshold of < 19.9, sensitivity was 84.2% and specificity 72.0%. For 41% SUVmax threshold segmentation, the TLG subset showed the highest AUC (0.717), with 75.0% sensitivity and 64.5% specificity for < 110.6 threshold. As for the grade 1 in liver pool threshold subset, compacity achieved highest AUC of 0.762 with 72.7% sensitivity and 82.7% specificity for < 1.478 threshold.

When separating grade 2 tumours, different parameters showed relatively higher value in different segmentation subsets. Using ITK-SNAP segmentation, LGZE achieved an AUC of 0.686 with 92.1% sensitivity and 38.1% specificity (> 0.004 threshold). Using liver pool segmentation, SRE achieved an AUC of 0.690, with 75.9% sensitivity and 55.0% specificity (< 0.941 threshold). Using 41% SUVmax segmentation, entropy achieved an AUC of 0.664 with 37.5% sensitivity and 86.8% specificity (< 1.225 threshold). Using SUVmax with 2.5 threshold segmentation, LGZE achieved an AUC of 0.680, with 45.1% sensitivity and 82.8% specificity (> 0.012 threshold).

When analysing the ability of individual parameters to identify grade 3 tumours, more homogeneous results were achieved. For all four different segmentation subsets, HGZE texture index achieved the highest value. The AUC of this parameter for ITK-SNAP, liver pool, 41% SUVmax, and SUVmax 2.5 threshold techniques was 0.717, 0.717, 0.715, and 0.713 respectively. Resulting sensitivities were 42.3%, 43.5%, 46.2%, and 47.2% respectively, while specificities were 96.2%, 93.4%, 91.4%, and 92.6% respectively. Corresponding calculated threshold values were > 386.7, > 272.5, > 509.4, and > 277.5.

### Multiparametric modelling and grade differentiation

Sensitivity, specificity, and overall accuracy obtained for each optimal selected model in all of four segmentation datasets for each tumour grade are shown in Table 2. The highest value to discriminate tumour differentiation grade was achieved in ITK-SNAP segmentation subset.

**Table 2:** Diagnostic accuracy of group method of data handling models to discriminate tumour differentiation grade in volumes from different segmentation techniques

Tumour differentiation grade	Segmentation techniques			
	SUVmax 2.5	Liver pool	41% SUVmax	ITK-SNAP
Model value (sensitivity %/specificity %/overall accuracy %)				
1	78.6/100.0/82.4	100.0/100.0/100.0	83.3/100.0/86.7	100.0/100.0/100.0
2	66.7/75.0/70.6	90.9/100.0/93.8	87.5/85.7/86.7	83.3/100.0/93.8
3	81.8/66.7/76.5	71.4/88.9/81.3	90.0/80.0/86.7	80.0/100.0/90.6
Model value (C-statistic/root mean square error/F-measure)				
1	0.818/0.457/0.778	0.989/0.244/0.943	0.961/0.288/0.921	0.976/0.244/0.946
2	0.672/0.606/0.618	0.928/0.349/0.875	0.940/0.335/0.887	0.975/0.233/0.988
3	0.672/0.679/0.540	0.967/0.321/0.892	0.955/0.301/0.901	0.944/0.216/0.991

*SUV* Standardised uptake value

## Discussion

Differentiation grade is an important biologic feature of malignant tumours, frequently incorporated into prognostication and influencing strategic decisions in patient management. Though being routinely identified during histopathological examination of biopsy samples or surgical material, noninvasive identification of tumour grade by means of semiquantitative and quantitative analysis of medical images represents interest in the context of combining radiomics with important clinical data. In the current study, we attempted to incorporate multiple semiquantitative and quantitative metabolic features into multiparametric modelling in order to try to differentiate tumour grades noninvasively.

In our dataset, we used multiparametric analysis by group method of data handling, incorporating all the extracted semiquantitative and quantitative features to create models that appeared to discriminate all three tumour grades in epithelial tumours with an overall accuracy ranging from 71 to 100%. It should be noted that these results were achieved for all four different segmentation technique datasets. The relatively most accurate model was achieved in subset with active contouring technique (ITK-SNAP segmentation).

Tumour segmentation in metabolic PET images is one of the most important steps in radiomics as it defines a group of voxels that are being assigned as representing active tumour tissue and all further mathematical manipulations for extraction of quantitative indices relies on these selected voxels. The segmentation is affected by various factors, both intrinsic and extrinsic, such as spatial resolution, noise level, shape, and location of pathologic tracer uptake [21]. Low spatial resolution of metabolic PET, especially, compared to anatomic imaging modalities, makes it difficult to define the precise tumour borders. Through recent years of research, a wide variety of segmentation or delineation techniques were proposed, including manual, thresholding-based, and boundary-based methods. Unfortunately, still no general agreement exists on optimal segmentation technique for PET radiomic studies.

First of all, different segmentation methods produce different values of quantitative parameters, mostly due to the inclusion or non-inclusion of necrotic tumour portions. The thresholding techniques with different cutoff values (SUV 2.5, relative thresholds of certain percentage of SUVmax, adaptive thresholding, for example Nestle’s method [22]) are ones that are more commonly used due to simplicity and intuitive and rapid workflow. Nevertheless, they are known to underestimate tumour volume and are susceptible to contrast variations, noise levels, and heterogeneity [23, 24].

In the current study, we used segmentation techniques offering different approaches. SUV 2.5 threshold technique was chosen as representing an “everyday practical” approach, as one of the simplest and less time consuming method, being easily incorporated into everyday practice. However, since SUV 2.5 threshold was first introduced in 2001, its clinical value was validated just for the solitary pulmonary nodule scenario [25]. Liver pool threshold technique was chosen in order to try to extend principles implemented in PET/CT imaging in lymphomas, being one of the reference sites in Deauville scoring system [26]. Forty-one percent SUVmax threshold technique was chosen as one of the methods suggested by EANM guidelines [13]. ITK-SNAP algorithm segmentation was chosen as representing an alternative, non-threshold approach, relying on three-dimensional active contour methods.

As theoretically expected, initial analysis of both semiquantitative and quantitative texture features showed that different techniques or thresholds to segment MTV results in significantly different values of this parameter.

It should be noted that there was no significant difference for all three conventional volumetric parameters between SUVmax with 2.5 fixed threshold technique and liver pool fixed threshold technique. This can be explained by the small difference between liver pool values (usually fluctuating between 2 and 3 SUV with our scanner, imaging protocol, and reconstruction algorithm) and SUVmax 2.5 threshold value.

Nevertheless, incorporating all extracted features into multiparametric modelling provided comparable ability of extracted data from different segmentation subsets to predict tumour differentiation grade.

Previously published studies have demonstrated how single semiquantitative PET parameters may be utilised to differentiate tumour grades, for instance in meningiomas [27], by means of tumour to grey matter ratio of ^18^F-FDG uptake (TGR). The TGR in high-grade meningioma (World Health Organization [WHO] grade II and III) was significantly higher than that in low-grade ones (WHO grade I) (*p* = 0.002) and significantly correlated with the MIB-1 labelling (cell proliferation marker) index (*r* = 0.338, *p* = 0.009) and mitotic count of the tumour (*r* = 0.284, *p* = 0.03). The ROC analysis revealed that the TGR of 1.0 was the best cutoff value for detecting high-grade meningioma with 43% sensitivity, 95% specificity, and 81%accuracy.

Dual-phase metabolic ^18^F-FDG PET approach with subsequent quantitative analysis was undertaken by Ghany et al. [28] in order to discriminate grading of gliomas. They found good correlation between the dual-phase PET grading and the histopathological grading of gliomas. When a 23% increase was used as the cutoff for analysis of the difference in SUVmax of the lesion *versus* normal grey matter over time, sensitivity was 88.9%, specificity 85.7%, and accuracy 89.4% (*p* = 0.003; AUC = 0.94). Nakamura et al. [29] investigated the connection between quantitative features of ^18^F-FDG uptake by endometrial carcinomas and International Federation of Gynaecology and Obstetrics (FIGO) grade: they found significant correlations between the SUVmax of the primary tumour and the FIGO grade, maximum tumour size, and glucose transporter-1 expression. Furthermore, multivariate analysis showed that the FIGO grade of endometrial cancer was most significantly identified as a relation factor of SUVmax (≥ 17.6). Rakheja and Probst [30] studied ^18^F-FDG uptake parameters for grading sarcomas and concluded that while ^18^F-FDG PET/CT cannot replace histopathology in the diagnosis of sarcoma, it is certainly of use in guiding surgeons and pathologists to biopsy the most aggressive regions of the tumour.

Similar to the above-mentioned studies, current one investigates connection of various metabolic PET parameters to tumour grade, but the most promising results are achieved when multiparametric modelling, utilising both semiquantitative and textural parameters, is applied.

Several limitations of the current study should be acknowledged. First of all, it was based on a single institution dataset, collected from one PET/CT scanner. Secondly, distribution of different tumour grades in our dataset is rather unequal, with grade 1 tumours comprising roughly only 10% of the sample. Consequently, these results should be further validated in a multi-institutional or multi-scanner scenario, in order to test whether the suggested model will withstand the difference in values of quantitative parameters extracted from images generated with different reconstruction algorithms, and on a larger patient population.

In conclusion, multiparametric modelling with GMDH utilising both semiquantitative and texture quantitative metabolic PET parameters seems to be an interesting tool for noninvasive malignant epithelial tumours grade differentiation. Results achieved in our dataset allow for hypothesise that, despite difference in absolute values generated by segmentation techniques, relative differences in combination of multiple parameters inside the subsets from different segmentation techniques allow to correlate with different tumour grades. Among extracted features, conventional semiquantitative and volumetric parameters demonstrated significant dependence from segmentation technique, while three quantitative texture indices remained stable. This allows to speculate that metabolic image features responsible for reflecting difference in tumour biology and grade are possibly more likely to be represented by heterogeneity of tracer uptake, rather than its intensity. Further investigation is required with larger patient population in order to validate the potential value of this approach.

## Data Availability

The datasets used and/or analysed during the current study are available from the corresponding author on reasonable request.

## Abbreviations

AUC: Area under the curve
FDG: Fluorodeoxyglucose
FIGO: International Federation of Gynaecology and Obstetrics
GLCM: Grey level co-occurrence matrix
GMDH: Group method of data handling
HGZE: Low and high grey level zone emphasis
LRE: Long-run emphasis
LGZE: Low grey level zone emphasis
MTV: Metabolic tumour volume
PET: Positron emission tomography
PET/CT: Positron emission tomography combined with computed tomography
ROC: Receiver operating characteristic
SRE: Short-run emphasis
SUV: Standardised uptake value
TGR: Tumour to grey matter ratio
TLG: Total lesion glycolysis
VOI: Volume of interest
WHO: World Health Organization
